# Burden of nosocomial rotavirus gastroenteritis in the paediatric population in Slovakia

**DOI:** 10.1186/2047-2994-4-S1-P226

**Published:** 2015-06-16

**Authors:** M Stefkovicova, Z Kristufkova, J Brnova

**Affiliations:** 1 School of Health Care, Alexander Dubcek University of Trencin, Trencin, Slovenia; 2 School of Public Health, Slovak Medical University, Bratislava, Slovenia; 3 Department of Laboratory Medicine, School of Health Sciences and Social Work, Trnava University, Trnava, Slovakia

## Introduction

Rotavirus gastroenteritis (RVGE) is most common nosocomial infection in paediatric department worldwide.

## Objectives

The aim of this study was assessed prevalence of nosocomial RVGE in children younger than 5 years in Slovakia during the last five years surveillance period.

## Methods

We assessed burden of hospital-acquired RVGE in Slovakia from national epidemiologic surveillance systems (EPIS) in period 2009 to 2013. Nosocomial RVGE was defined i) when the child was admitted with a diagnosis other than gastroenteritis ii) when the first symptoms of RVGE appeared not earlier than 24 h after admission iii) when the family reported no signs of diarrhoeal diseases iii) when the child was re-hospitalized at the children’s department within 3 days (incubation period for RVGE) with symptoms of gastroenteritis after the first admission iiii) when RVGE was confirmed by laboratory testing (ELISA or rapid immunochromatographic test).

## Results

RVGE was clinically and laboratory confirmed in 11 967 in children younger than 5 years. Each year were reported on average 2393± 576 (1803 – 3222) cases, of them 78,9 % required hospitalisation. According the criteria for nosocomial infection totally 1533 (12,8 %) cases were nosocomial RVGE. Additionally, in Slovakia immunisation of infants with rotavirus vaccines has been implemented since 2006 and vaccination coverage reached 17,5% in year 2013.

## Conclusion

Nosocomial RVGE represents a serious epidemiological and economical problem in Slovakia. Mandatory vaccination covered by health insurance and better practise in hospital hygiene, especially improvement in compliance to multimodal strategy for hand hygiene, could reduce prevalence of nosocomial RVGE on paediatric department in Slovakia in the next decades.

## Disclosure of interest

None declared.

